# Principles and Methods of Filling Teeth with Gold

**Published:** 1886-05

**Authors:** A. G. Bennett

**Affiliations:** Philadelphia, Pa.


ARTICLE II.

### PRINCIPLES AND METHODS OF FILLING TEETH WITH GOLD.

BY A. G. BENNETT, D. D. S., PHILADELPHIA, PA.

To the question, "What is the most urgent need of dentistry to-day?" several answers may be given. Perhaps few will dispute the assertion that our great need is scientific knowledge—exact and exhaustive—of the structures and materials, the pathology and therapeutics, involved in saving teeth. No one doubts that "the need of the profession of the age is to know;" but that "our mechanical ability has outrun our scientific attainments," may be considered a debatable question. To what extent have we been able to utilize our knowledge of dental anatomy and physiology in saving teeth? It is true that our most successful operators have usually been, and are, the best educated; yet skill, after all, is the one thing needful to give any meaning or value to knowledge in practical dentistry. Dr. Thompson claims that "it is most imperative and important that we better understand *why* we perform our operations; the *how* will follow of itself. Filling a hole in a tooth is an absurdly simple thing to do—a mere mechanical and artistic performance." The *why* we fill a tooth has never been considered much of a mystery. The tooth cannot resist decay nor restore the lost portion—in short, it cannot fill itself. Again, what is the value of "scientific reasoning," as opposed to experimentation? Is it not a fact that all theories and supposed principles are established or destroyed by experimentation and experience? Again, Dr. T. claims that we are not scientific, because the lost parts are not restored in natural substance. There may be such a thing as "striving after the unattainable."

As long as the operation of filling a tooth is slightly surgical and largely mechanical, so long there will exist a greater necessity for developing skill than for acquiring knowledge. How many educated dentists are guided in operating by the structure and functions of the dental tissues? The enamel-rods, the living fibres, the nutritive currents, receive little or no attention, or must yield to mechanical requirements.

When all is summed up, it must be admitted that, though one *should* have the learning, he *must* have the skill. It has been not unfrequently more than hinted that some operators who can talk fluently and write elegantly on saving teeth are unable to make even a presentable filling.

Filling a hole in a tooth may be an absurdly simple thing as regards mechanical principles, especially if the tooth is to be thrown into the operator's drawer; and simple enough, even when the tooth is exposed to the forces and fluids in the mouth, if the object be merely to insert the filling so that it will be retained till it decays out; but to insert a gold filling in such a manner that the tooth will resist, for say ten years, all chemical, thermal, and perhaps electrical, forces to which it may be exposed, is quite a different thing.

Our text-books on operative dentistry, though treating the subject of filling in detail, give space and importance to certain features of the subject out of proportion to their relative value. For instance, Taft dwells at great length on the preparation of cavities by classes and their modifications, and on the various forms of gold; yet gives comparatively little exact information and few definite directions on the most essential of all points—the adapting or packing of the gold against the walls and around the margins of the cavity. No one doubts or denies the fact that proper preparation of the cavity is the basis of a perfect filling; but, after all, the adaptation of the gold to the dentine and enamel surfaces is the essential requirement. This is trite enough to those who use gold successfully, but it has not received the general recognition which its importance demands. Adaptation is the vital point, for the obvious reason that defects, even the smallest in the cavity, can readily be seen, with or without a magnifier; and removed, while defects in packing the gold are more or less concealed from view, and generally cannot be corrected except by taking out all or a part of the filling.

Some one remarks that it is difficult, if not impossible, to adapt two hard substances such as dentine and extra-cohesive gold to each other so as to form a moisture-proof joint; hence the necessity of having as much softness in the gold as is consistent with the required cohesion, and hence the necessity of an even, smooth wall; for, since adaptation to such a wall is difficult, it is obvious that it is well-nigh impossible to adapt gold against rough, uneven surfaces. It is clearly impossible to force gold into the minute inequalities of dentine and enamel. In short, as some one has said, a filling should resemble a cork in a bottle rather than a ground-glass stopper.

Though the essentials of successful tooth-filling are more or less familiar to all, yet, as a basis for what is to follow, they will bear repetition. "It has been said," remarks Dr. Atkinson, "that almost anybody can make a filling moisture-tight. Almost nobody does. If the cavity is properly prepared, you will have no difficulty." The expert few may have no difficulty, but the unskilled many, even with a perfectly prepared cavity, will often fail of success.

To prevent breakage and leakage, and because a tooth is partly an animal tissue, and not wholly a mineral substance, the following are the essentials for cavity preparation:

1. A cavity should be so prepared and its border so beveled that when filled the tooth will offer the greatest resistance to mechanical and chemical forces.

2. Complex cavities should be so simplified and their parts made so accessible that the filling material can be readily and certainly adapted to their walls.

3. Approximate cavities, which extend to the excising edges or occluding surfaces of the teeth, should be so prepared and filled that the strength of the operation will be equally divided between the tooth and the filling.

4. The walls of a cavity should have no corners or acute angles, and should, when possible, form the segment of a circle; and the bevel of the enamel should, as far as may be, conform to the line of its cleavage.

5. Smooth, strong walls, secure anchorage, and perfect adaptation of the filling material to the tooth-bone, are the essentials of durability.

6. As regards the enamel, it is better to remove too much than too little; as respects the dentine, better to remove too little than too much; and as to the anchorage, it had better be too deep than too shallow.

7. Anchorage should be secured by so combining pits and grooves as to do the least injury to the dentine and give the greatest strength to the filling; and the enamel should, when possible, be supported by living dentine.

And, to sum up, smoothness of surface and softness of material insure closeness of adaptation.

A few words on the final preparation of approximal cavities may not be amiss. Dr. Jack's idea that a grove-cutter should have a rounded edge, is certainly a good one. A concave floor to the groove gives greatest strength, besides being exactly adapted to a convex surface on the plugger. After grooving, take a sharp spoon excavator of the proper size, and remove the fine edge from the margin of the groove. With a chisel excavator cut and scrape the cervical wall till all softness of the dentine and roughness and whitish appearance of the enamel have been removed; then polish all accessible surfaces.

Much as opinions and theories may differ in regard to the various forms and methods of using gold, all successful operators are agreed that softness of the metal is the essential property for tooth-preservation. The value of the cohesive property, essential as it is for contour work and in restoring crowns, has been over-estimated, and to many it has proved a delusion and a snare, because of easy welding and difficult adaptation. Who has not observed and admired the beautiful working of this gold? Yet how few have noted the fact that its adaptation could scarcely be worse. To many for years the cohesive property contained the "promise and the potency" of all that is "ideal" in the perfect filling material. For those who claimed that if a tooth was worth filling at all it could be filled with gold, and then proceeded to fill all cavities without regard to the quality of the tooth-bone with cohesive gold, there could be nothing but ignoble failure. Some few expert manipulators of cohesive gold have attained phenomenal success; but of some of these one can truly say: "they did not live to see their wrecks," their careers being cut short by too intense devotion to their golden idol.

If there was nothing more difficult in operative dentistry than the welding of cohesive foil, even when one end of the mass is anchored in a carious cavity, the record of failures would form a small part of dental literature. Those who have regarded the cohesive property as something of a marvelous mystery, seem to have lost sight of the two facts that cohesion is an inherent property of all metals, and that gold when pure and clean welds cold simply because it does not oxidize. Had the value of softness in cohesive gold been noted and understood sooner, there would be less reason to be lost in wonder that teeth decay so readily and so rapidly around gold which worked so beautifully. True, cohesive gold is relatively soft and ductile, and when used in the form of ribbons, even though made from heavy foil, some operators have no doubt been able to "swage" and "strap" and "band" it beneath and over and around the frail enamel walls of even delicate laterals; but the inherent ability and acquired skill to do these things have been given to the favored few.

The best gold is not always "that which can be worked the most easily, rapidly and perfectly," but that which can most safely and certainly be adapted to dentine and enamel walls. Extra-cohesive gold may not work the most easily, but it certainly works the most rapidly, as large pieces and many thicknesses can be used under a broad plugger; and it works perfectly, since it makes a mass which is securely welded; yet the adaptation may be most defective.

Not many will dispute the assertion that few gold fillings are absolutely perfect. Most of them have two kinds of defects—visible and invisible; the former of course being around its margins in the enamel or gold, and the latter at any point over the entire surface of the cavity. As the condition of the cavity can be readily seen and the kind and form of gold selected, the causes of these defects can be narrowed down to the instrument and its proper or improper management.

1. It may be laid down as an axiom that pluggers should be so constructed and gold used in such a manner as to exclude, with the minimum of time and attention, all defects which from their nature cannot be detected by the eye.

2. Not only the shape of the tooth, but mechanical requirements, as well as esthetic considerations, demand that the walls of a cavity should generally form the segment of a circle. And if their form be not a matter of mere fancy, the size and shape of end and curve of shank in filling instruments must be determined by and adapted to the size and shape and position of the cavity.

3. Owing to the position and condition of cavities and teeth, gold is adapted to dentine and enamel surfaces *directly or indirectly.* When the walls are strong enough it should be adapted directly, and when the walls are weak or the tooth frail it must be adapted indirectly—or, in other words, spread or wedged against the walls. This determines that there are essentially two kinds of pluggers—one for direct, the other for indirect, adaptation.

4. The cavity walls, especially the cervical, being concave, requires that the face of a "direct" plugger should be convex, to secure perfect adaptation; and this form of face, besides, will insure thorough condensation and welding more certainly than a flat surface, because of the difficulty of holding two flat surfaces squarely against each other.

5. Experiments prove that gold will spread only under a convex surface, either smooth or serrated transversely, the instrument when serrated being so held that the cuts or valleys are at right angles to the cavity wall. Pits and grooves, as well as small cavities and fissures, admit only of indirect adaptation; hence "indirect" pluggers for these should always have a convex face, serrated in one direction only, so as to spread the gold slightly but firmly against the walls. "Direct" pluggers, when used within the cavity, may be serrated one or both ways; but when used beyond the wall they should be serrated in both directions, for the reason that here spreading of gold would be a detriment rather than an advantage.

6. "Direct" pluggers are generally of the foot shape, and consist of two kinds—one short and relatively thick, and the other long and thin; the short being used within the cavity and beyond its walls, as in restorations, and the long being intended to complete the filling where space is limited, as between the front teeth. The end of an "indirect" plugger is usually small, and may be round, oval or flat.

7. In regard to serrations, it may be observed that they answer two objects, viz.: prevent slipping, and leave a rough surface. Smooth points, though they spread the gold, have been tried and found wanting, because by slipping slightly they burnish the gold, thereby damaging, if not destroying, the cohesive property.

8. Smooth convex points, besides spreading the gold, prevent pitting and porosity. Small points serrated both ways effectually prevent spreading, besides tearing and cutting up the gold; but convex surfaces cut one way combine the good qualities of both kinds without the defects of either. When soft gold is packed like tin, of course the serrations must be deep, and the plugger should be cut both ways, so as to secure sharp points to interlace the metal; but when the cohesive property is utilized, no one will now claim that the serrations are intended to cut through and interlock the gold.

9. The pitting or porosity caused by serrations is reduced to the minimum by having the pluggers cut fine. As pluggers of the foot shape are seldom available for indirect adaptation, and because of their relatively large surface do not cut up the gold, they should always be serrated in both directions; but no good reason can be given why a small "indirect" plugger should be cut both ways.

The writer has devised a set of pluggers based on the foregoing principles and theories, which he has endeavored to verify. These points are the result of a number of experiments undertaken with the view of determining the best form of face and kind of serrations for making a moisture-proof joint with the least risk of injury to the tooth-bone. These points are so shaped and serrated that some, if not all, of the common and usually invisible defects can be excluded with a good degree of ease and certainty, and that, too, without unduly taxing the time and attention of the operator.

This set of pluggers consist of modified and original forms. There are five ordinary round and one flat point, and six foot-pluggers, three short and three long; and it is believed that these, in connection with the most useful hand-pressure points, will enable the operator to reach every cavity that can be entirely or partly filled by mallet force. This set of instruments was illustrated in the *Dental Cosmos* for October, 1885.

A few words on the automatic mallet, for which these points are intended, may not be amiss. The blow of the Snow & Lewis automatic mallet, which is justly regarded as the best, is a little too sharp and painful for the safety of the enamel and comfort of the patient. These defects are easily removed. It has been the habit of the writer for some years to put a drop or two of castor-oil on the end of the mallet, which is done by taking it out of the case. The blow is modified partly by some of the oil remaining on the upper end of the plugger-socket, and partly by some of it gradually working in around between the mallet and the case, thereby retarding the descent of the former and increasing its effectiveness; for the blow seems to combine all the good qualities of steel and lead without their objectionable features. Besides this, the working parts of the mallet should be occasionally lubricated with engine-oil. Owing to the strength and temper of the spring, all mallets cannot be equally modified by the method just described. To save time and avoid changing points, the operator should have two or three automatic mallets, or it should be used in combination with the hand-mallet.

In concluding, the writer would say that, though these points may not be perfect as made, it is believed that it can be demonstrated that they embody the correct principles.—*Dental Cosmos.*

